# Cuckoo-PC: An Evolutionary Synchronization-Aware Placement of SDN Controllers for Optimizing the Network Performance in WSNs

**DOI:** 10.3390/s20113231

**Published:** 2020-06-06

**Authors:** Shirin Tahmasebi, Mohadeseh Safi, Somayeh Zolfi, Mohammad Reza Maghsoudi, Hamid Reza Faragardi, Hossein Fotouhi

**Affiliations:** 1 Department of Computer Engineering, Sharif University of Technology, Tehran 11365-11155, Iran; shtahmasebi@ce.sharif.edu; 2 Shariaty Technical College, Technical and Vocational University, Tehran 13114-16846, Iran; mhds.safi@gmail.com; 3 School of Computer Engineering, University of Science and Technology, Tehran 16851-18918, Iran; 4 Zand Institute of Higher Education, Shiraz 71887-73489, Iran; mr.maghsoudi88@gmail.com; 5 School of Electrical Engineering and Computer Science, KTH Royal Institute of Technology, 100 44 Stockholm, Sweden; 6 School of Innovation, Design, and Engineering, Mälardalen University, 721 23 Västerås, Sweden; hossein.fotouhi@mdh.se

**Keywords:** wireless sensor networks, software defined networks, controller node placement, Cuckoo optimization algorithm, synchronization cost

## Abstract

Due to reliability and performance considerations, employing multiple software-defined networking (SDN) controllers is known as a promising technique in Wireless Sensor Networks (WSNs). Nevertheless, employing multiple controllers increases the inter-controller synchronization overhead. Therefore, optimal placement of SDN controllers to optimize the performance of a WSN, subject to the maximum number of controllers, determined based on the synchronization overhead, is a challenging research problem. In this paper, we first formulate this research problem as an optimization problem, then to address the optimization problem, we propose the Cuckoo Placement of Controllers (Cuckoo-PC) algorithm. Cuckoo-PC works based on the Cuckoo optimization algorithm which is a meta-heuristic algorithm inspired by nature. This algorithm seeks to find the global optimum by imitating brood parasitism of some cuckoo species. To evaluate the performance of Cuckoo-PC, we compare it against a couple of state-of-the-art methods, namely Simulated Annealing (SA) and Quantum Annealing (QA). The experiments demonstrate that Cuckoo-PC outperforms both SA and QA in terms of the network performance by lowering the average distance between sensors and controllers up to 13% and 9%, respectively. Comparing our method against Integer Linear Programming (ILP) reveals that Cuckoo-PC achieves approximately similar results (less than 1% deviation) in a noticeably shorter time.

## 1. Introduction

Software defined networking (SDN) [1] provides fine-grained information to select the best forwarder to form global resource optimization rather than ad-hoc networks. This network technology provides the possibility of network reconfiguration through on-the-fly programming [2]. Recently, using SDN in Wireless Sensor Networks (WSNs) has become an increasingly important trend. Due to the high dynamicity of WSNs, using SDN in WSNs can provide several benefits, including improving flexibility, boosting scalability, eliminating the complexity of the network, better configuration and management, and replacing rigidity to policy changes [3,4]. Dynamic reconfiguration of the network is a useful feature, especially in harsh environments, where wireless links could be highly unreliable, and thus network routing should be updated frequently [3,5]. Moreover, large scale WSNs will require multiple SDN controllers in order to manage their configuration.

It is worth mentioning that due to different characteristics between WSNs and traditional wired networks, applying SDN in WSNs introduces several new challenges, including [6]:

- In a WSN, most of the devices face severe limitations in terms of processing power, storage, and battery resources. Hence, it is of paramount importance to reduce unnecessary packet transmission in SDN-enabled WSNs in order to reduce packet collision and extend network lifetime. However, in a wired network, the main goal is to minimize the response time.
- Unlike wired networks, the links in a WSN are highly unstable and unreliable. Therefore, WSN sensors are subject to several types of failures, namely, communication failures due to environmental obstacles and power failures due to constrained and insufficient battery resources. Thus, to overcome link unreliability, multipath solutions may be applied to increase the chance of successful data transmission that, in turn, will increase network overhead.

Accordingly, it can be concluded that the nature of WSNs is significantly more dynamic than wired networks. Hence, the controller placement in WSNs, in comparison to wired networks, is more critical and challenging and has a more profound impact on the performance, lifetime, and Quality of Service (QoS) of the network.

Since the SDN controller demands high storage and large computational power, the limited computational and storage capacity of available WSN devices hinders the implementation of the SDN controller. A promising solution to implement the SDN controller is to integrate additional node(s) within WSNs, which are supposed to be responsible for executing the SDN controller software. Although the notion behind the control plane in software-defined networks is based on a centralized fashion, the SDN controller must be physically distributed among multiple nodes to achieve higher performance, scalability, and fault tolerance [7,8].

If we adopt a distributed version of the SDN controller in WSNs, then the question is how to deploy the SDN controller nodes in the network. How many nodes are required, and where should they be placed? Two important QoS elements should be taken into account when it comes to the deployment of multiple controller nodes in a WSN, namely, (i) reliability and (ii) latency.

Reliability is a challenging issue in WSNs since low-power links are highly unreliable, and the link quality fluctuates a lot [9]. Link failure can cause node inaccessibility to the network, disconnections, network performance degradation, and eventually node failure. In SDN-enabled WSNs, it is highly important to guarantee wireless connection to SDN controllers as they are acting as the brain of the network, and provide network reconfiguration. Thus, it is crucial to provide network reliability by replicating SDN controllers in order to avoid a single point of failure [10]. Hence, in the network architecture, multiple controllers should be accommodated to achieve a higher reliability.

Latency is an important performance metric that can be considerably affected by the placement of controller nodes. A tactful placement of controller nodes can improve the network performance by reducing the distance between sensor nodes and the controller(s) covering the sensors. A shorter distance between controllers and sensors also decreases the network traffic and saves energy consumption. Thus, one of the major parameters for placing controllers in a sensing area is the distance to sensor nodes.

The physically distributed control plane in SDN-enabled WSNs could result in more reliability, fault tolerance, timeliness, and better performance. However, to make the control plane logically centralized, it is necessary to synchronize controllers and provide a consistent view of the network’s state for all of them. Thus, a physically distributed control plane can provide such benefits at the expense of synchronization costs. In [7], the trade-off between the synchronization cost of multiple controllers and network latency was discussed. The results indicated the feasibility of multi-controller deployments in terms of network latency if the right number of controllers are deployed.

The efficient node placement in SDN-enabled WSNs has essential benefits in several application domains. For example, it can be used in industrial applications, such as smart factories, to enable environmental monitoring, enhance productivity, increase flexibility, improve energy consumption, and reduce maintenance costs. In such harsh environments, where performance and time sensitivity are of paramount importance, using a single controller to manage the whole network is not sufficient. Instead, it is more effective and also challenging to deploy multiple SDN controllers to ensure the satisfaction of requirements [11,12,13,14].

In this paper, we intend to find an optimal controller placement to maximize the network performance subject to (i) a certain upper bound for the synchronization cost that limits the maximum number of controllers, and (ii) reliability constraints. We formulate the problem as an optimization problem, which is then modeled as an Integer Linear Programming (ILP). The proposed ILP model is solved using the *Cuckoo Placement of Controllers (Cuckoo-PC)* algorithm and the results are compared not only with the recently proposed methods in the literature, but also with the CPLEX ILP Solver.

**Contributions.** Our major contributions are listed as follows:

1. Specifying the optimal placement of controller nodes in a WSN to optimize the network latency with respect to the inter-controller synchronization overhead.
2. Targeting both reliability and performance (in terms of the number of hops between controllers and sensor nodes) in the deployment phase of SDN-enabled WSNs.
3. Proposing the Cuckoo-PC algorithm to solve the placement optimization problem, which considerably outperforms ILP and Quantum Annealing (QA) in terms of scalability and performance, respectively.

Organization of the paper: in Section 2, a comprehensive review of related work is presented. In Section 3, we describe the problem and assumptions, following by formulating the problem as an optimization problem. Section 4 presents the algorithm proposed to address the optimization problem. In Section 5, the performance of the proposed method is investigated. Finally, in Section 6, we conclude our paper by providing a summary along with the future directions.

## 2. Related Work

Several works have addressed node placement in WSNs and IoT systems [15,16,17]. These studies concentrate on different types of nodes and different performance metrics in their node placement strategies. The authors in [18], investigated the sensor placement problem and focused on two metrics: coverage and connectivity. They formulated the problem as a constrained optimization problem, and proposed two heuristic algorithms, named the Connected Cover Formation (CCF) and the Cover Formation and Relay Placement with Redundancy Removing (CFRP-RR).

### 2.1. Sink Placement in WSNs

The authors in [19] investigated sink and relay node placement in WSNs. They presented several algorithms, named as Greedy-MSRP and GRASP-MSRP. Greedy-MSP and GRASP-MSP, to optimize the deployment cost and to guarantee reliability. The authors in [20], investigated multiple sink placement in WSNs and proposed a novel method based on Genetic Algorithm (GA) to minimize the worst-case delay and energy consumption. Multiple sink placement problem has also been explored in [21]. In [21], the authors presented a Particle Swarm Optimization (PSO) based approach to minimize the number of required sinks and the path length between sensors and sinks. In [22], optimal sink selection and placement in WSNs is addressed using a PSO-based algorithm. Their main focus is to prolong the network lifetime, improve energy efficiency, and minimize intra-cluster distance in terms of hop count. In [23], an approximation algorithm, called GREEDY-k-SPP, is proposed to solve multiple sink placement problem and minimize the worst-case delay. In [24], a comparison was made to evaluate the effects of static and dynamic sink node placement on several QoS parameters such as packet delivery ratio, average throughput, and average end to end delay. Their results revealed that all of these parameters were improved in the dynamic sink placement.

### 2.2. Controller Placement in Wired Networks

The controller placement problem for wired networks has been addressed in several papers. In [25], the authors explored the trade-offs for optimizing the minimum latency between nodes and controllers. Hock et al. [26] is an extension of [25], which was referred to as Pareto-Optimal Controller placement (POCO), considering additional aspects other than network latency. Feixiang Li et al., in [27], addressed the controller placement problem in wired networks to minimize the latency between nodes and controllers. They formulated the problem as an ILP and proposed three heuristics to solve it: Cuckoo Search Algorithm (CSA), Genetic Algorithm (GA), and Particle Swarm Optimization (PSO). Their experiments revealed that although CSA processing time is longer than the other two algorithms, it has the best performance among two others in terms of average latency between nodes and controllers.

In [28], the authors focused on establishing a survivable control plane for SDN networks by using a novel mutual backup model. Moreover, they formulated the problem as an ILP model and designed a heuristic approach. In [29], the authors focused on the security aspects of SDN networks and proposed a solution to enhance network resiliency. One of the most critical failures which can negatively affect the fault tolerance of the network is Byzantine attack, wherein the malicious node can either stop responding or continue to generate arbitrary data, pretending to be correct. Hence, to guarantee the proper update of flow tables in each switch, it is assumed that each switch in the network has communication with multiple controllers that run the BFT protocol.

### 2.3. Controller Placement in WSNs

In WSNs, [30] presented a deployment strategy for multiple controller nodes that minimizes the transmission delay of the network while satisfying different critical requirements such as deployment cost and reliability. In [31], the controller placement problem was investigated in a wireless SDN. The main metrics considered in these works were: (1) link failure probability, (2) average throughput in southbound interface (3) transparency, which is introduced as the latency caused by interference from controllers, and (4) latency. Although the Euclidean distance is usually used to measure latency, in [31], a much more complex model was used to reflect the effects of collision and interference on latency. Finally, hill climbing with simulated annealing was used to find an efficient solution. Qin et al. in [32] studied the controller placement problem in edge networks. Since increasing the distance between controllers and nodes increases delay significantly, a scattered placement of controllers across the network would reduce both the distance and delay. However, scattered placement may increase controller synchronization overhead. Thus, delay and controller synchronization overhead are two contradicting objectives. On the other hand, in their proposed model, two strategies were considered for controller synchronization: (i) leaderless, and (ii) leader-based. Finally, the optimization objective was to minimize delay and controller synchronization overhead for both strategies. In [33], the controller placement was divided into two problems. The first is how to select controller positions and the second is how to allocate switches to each controller to optimize delay and reliability. Thus, two meta-heuristic algorithms were used to solve these problems: (i) Louvain algorithm to solve the first problem and (ii) PSO to solve the second one. Alenazi et al., in [34], aimed to solve the controller placement problem, considering network resilience and delay performance. A novel node metric was introduced, which was called nodal disjoint path (NDP) that measured node’s importance in terms of its path diversity to other nodes. Moreover, two greedy algorithms were used to solve the selection problem: (i) NDP-global and (ii) NDP-cluster. Due to the fact that using a centralized control plane may incur a bottleneck, in [35], a two-level hierarchy control framework was introduced to have a decentralized architecture. These two layers were called master and slave, and the goal was to optimize controller placement in the slave layer. The optimization objective was to minimize control delay and control cost of critical nodes. Node criticality was evaluated by Fuzzy AHP based on several criteria and factors: device attributes (type, location, and owner), service attributes (delay requirements, privacy requirements, the service user, and service profit), and control frequency (number of communications with the controller at one given time). Finally, this optimization problem was solved by Particle Swarm Optimization (PSO) algorithm.

### 2.4. Investigation of Inter-Controller Synchronization Overhead

The inter-controller synchronization costs have been investigated in several studies. In [7], the authors focused on this problem and used two metrics to measure the inter-controller synchronization cost: synchronization delay (the time since a controller generates an event until a different controller is aware of that same event) and the amount of synchronization data exchanged between controllers. Their experiments revealed that the synchronization delay is insignificant in most cases, and it is feasible to trade off inter-controller synchronization cost for more fault tolerance and scalability. In [36], the authors proposed a framework for dynamic controller deployment and provisioning. They used several metrics to measure deployment cost: statistics collection costs, flow setup costs, inter-controller costs, and reassignment costs. The problem is then formulated as an ILP, and two heuristics are proposed: First, a greedy approach based on the knapsack problem (DCP-GK) and second, a meta-heuristic approach based on simulated annealing algorithm (DCP-SA). In [37], several metrics are considered to address the controller placement problem: inter-controller synchronization cost, controller-switch communication cost, flow statistics collection cost, and measurement overhead. Finally, two algorithms are proposed to approximate the solution: Discrete Approximation Algorithm (DAA) and Connectivity Ranking Algorithm (CRA). Their experiments revealed that the proposed method could reduce the measurement overhead by 40% on average. The authors in [38] solved the controller placement problem by focusing on the minimization of the total required bandwidth for the inter-controller synchronization traffic and also represented experimental results in a realistic environment that was offered by an SDN testbed.

## 3. Problem Modeling

This paper addresses an SDN-enabled sensor network, where sensor nodes collect data and forward it either directly to a sink node (if the sink node is located in the coverage area of the sensor) or towards a sink node by handing the data to the neighbor sensors. It is assumed that the placement of the sink nodes has already been accomplished using state-of-the-art methods such as [8,11], and our paper mainly focuses on the placement of controller nodes respecting the given location of sinks and sensors.

### 3.1. Problem Representation

A WSN is represented by an undirected graph, where vertices are partitioned into a set of sensors *T* that continuously generate data, sinks *S* to collect the sensors’ data, and controllers *C* to implement the SDN functionalities. Consequently, in the graph representing a WSN, V=T∪S∪C. An edge of the graph denotes a wireless connection between a pair of nodes. The total number of sensors is *N*, where $N=\left|T\right|$. A pair of nodes connected with an edge is called *neighbor*. Furthermore, to represent the placement problem, we define *A_C_* as a set of candidate controllers. Hence, $C\subseteq A_{C}$.

To represent the problem, a binary vector *X* is utilized which determines whether a candidate controller has been selected or not. Thus, the size of *X* equals to the number of candidate controllers:

(1) $$X_{i}=\left\{\begin{array}{cc} 1 & \mathrm{if}\;\mathrm{the}\;\mathrm{candidate}\;\mathrm{controller}\;i\;\mathrm{is}\;\mathrm{chosen}\\ 0 & \mathrm{else} \end{array}\right.$$

### 3.2. Reliability Constraints

A sensor is *k*-controller-covered if and only if it has at least *k* paths of length $\leq l_{max}$ to *k* controllers in *C* (*k* is an integer number ≥ 1). A network is defined as *k*-controller-covered if each sensor v∈T is *k*-controller-covered. In order to represent the *k*-controller-covered constraint, we define the binary matrix *Y* that determines whether the shortest distance between the *i*th sensor and the *j*th candidate controller is shorter than $l_{max}$ or not:

(2) $$Y_{i,j}=\left\{\begin{array}{cc} 1 & l^{*}\left(v_{i},c_{j}\right)\leq l_{max}\\ 0 & \mathrm{else} \end{array}\right.$$

where $l^{*}\left(v_{i},c_{j}\right)$ is the shortest path (in terms of the number of hops) between node $v_{i}$ and controller $c_{j}$, which can be calculated by the Dijkstra algorithm.

It is expected that each sensor is covered by *k* controllers (*k* ≥ 1) to avoid a single point of failure and to have a reliable network. Now we can formulate the *k*-controller-covered constraint as follows:

(3) $$\sum_{\forall c_{j}\in A_{C}}Y_{i,j}X_{j}\geq K;\forall v_{i}\in T$$

where $v_{i}$ denotes the *i*th sensor.

### 3.3. Timing Constraints

To meet timing requirements in WSNs, the rate of routing requests coming from sensors to a particular controller should not exceed a threshold. The threshold denotes the maximum rate of routing requests that a controller can process [31,39] in an acceptable time. In other words, if the load of a controller exceeds the threshold, the controller is overloaded. This threshold is considered in our model and called as the load constraint. In the formulation of the load constraint, the load of sensor *i* is denoted by $\omega_{i}$ and defined as the rate of routing messages per second for those routing messages that do not match the sensor’s lookup table and must be sent to the controller [31]. If a sensor covered by *n* controllers (i.e., the distance between those *n* controllers and the sensor is shorter than $l_{max}$), the load of the *i*th sensor on the *j*th controller covering the sensor is denoted by *w_i,j_*, which is equal to $\omega_{i}/n$. In other words, we assume that the load of a sensor is uniformly distributed across the controllers covering the sensor [11]. Let us assume that all the controllers have the same type and the same capacity, and *W* shows the maximum workload that a controller can handle within an acceptable time, then the workload constraint is reflected by:

(4) $$\sum_{\forall v_{i}\in T}Y_{i,j}X_{j}\frac{\omega_{i}}{\sum_{\forall c_{t}\in A_{C}}Y_{i,t}X_{t}}\leq W/\left(k-1\right);\forall c_{j}\in A_{C}$$

where $\sum_{\forall c_{t}\in A_{C}}Y_{i,t}X_{t}$ is the number of selected controllers covering the *i*th sensor. Due to reliability requirements, we apply a more strict workload constraint; if k−1 controllers fail, the remaining active ones should be able to handle the entire workload of all the failed controllers without any violation of the latency requirements. That is the reason to divide the right side of the inequality by k−1.

It is worth mentioning that besides the load constraint, the limitation of the number of hops between sensors and controllers reflected in the definition of *k*-controller-covered is a sort of timing constraint; otherwise, having only *k* controllers would be enough even for huge WSNs.

### 3.4. Inter-Controller Synchronization Cost

To calculate the inter-controller synchronization cost, we use a function called as *SyncCost^Controller^($\left|C\right|$)* which is a function of the number of selected controllers. The more the number of controllers, the higher the value of this function. To calculate the value of this function for the different number of controllers, we use the flow table synchronization overhead introduced in (Figure 10 of [32]), where the inter-controller synchronization cost is investigated and classified into several types.

In addition, a predefined limit for the maximum inter-controller synchronization overhead with respect to the size of the network is considered, denoted by *SyncLimit(N)*. Indeed, *SyncLimit(N)* is a function of the number of nodes in the network and is a non-descending function growing with the size of the network. In other words, for bigger networks, this limit increases. To calculate the value of this function, we use the results presented in (Figure 3b of [32]), where controller-node synchronization overhead for different sizes of the network is investigated. Apparently, if too many controllers are selected such that the inter-controller synchronization overhead exceeds *SyncLimit(N)*, the synchronization overhead can hinder further improvement of the network performance.

Therefore, to respect the synchronization overhead constraint, the number of selected controllers, i.e., |C|, is set to the maximum value that satisfies the following condition:

(5) $${SyncCost}^{Controller}\left(|C|\right)\leq SyncLimit\left(N\right),\;1\leq |C|\leq \left|A_{C}\right|$$

The reason to consider a maximum value for selected controllers is that a higher number of controllers can potentially improve the network performance, as long as the condition of Equation (5) holds. Therefore, the overall size of the problem space is turned to $Combination(|A_{C}|,|C|)$ which is equal to the number of combinations to choose |C| out of $\left|A_{C}\right|$.

Obviously, when the inter-controller synchronization limit is high enough to choose all the candidate controllers (i.e., $\left|C\right|=\left|A_{C}\right|$), the problem can be simply solved by choosing all the candidate controllers. However, most often, ${SyncCost}^{Controller}\left(\left|A_{C}\right|\right)>SyncLimit\left(N\right)$, implying that selecting all the candidate controllers leads to violation of inter-controller synchronization constraint.

Hence, the constraint that we need to consider in our optimization model is

(6) $$\sum_{\forall c_{j}\in A_{C}}X_{j}=\left|C\right|$$

where |C| is calculated according to Equation (5).

### 3.5. Optimization Problem

**Objective.** Since the controller needs to keep in touch with sensors to manage the routing decisions dynamically, the number of hops between sensors and the controller(s) considerably affects the network performance. A long path between sensors and the controller(s) can increase not only the network traffic but also the delay of exchanging control messages. Accordingly, we would like to place the controller nodes such that the farthest controller that covers a sensor becomes as close as possible to the sensor. Accordingly, to minimize the maximum distance between sensors and controllers, we should:

(7) $$Minimize:\;\;\;\max_{\forall v_{i}\in T}\left\{L_{v_{i}}^{*}\right\}$$

where $L_{v_{i}}^{*}$ indicates the distance between $v_{i}$ and its furthest controller among the *k* controllers covering the sensor and formulated as follows:

(8) $$L_{v_{i}}^{*}=\max_{\forall c_{j}\in A_{c}}\left\{Y_{i,j}X_{j}l^{*}\left(v_{i},c_{j}\right)\right\}$$

Accordingly, the optimization problem is formulated as follows:

(9) $$\begin{array}{c} Minimize:\;\;\;\sum_{\forall v_{i}\in T}\left\{L_{v_{i}}^{*}\right\}\\ Subject\;to:\;\;\;(3),(4),(6) \end{array}$$

### 3.6. An Illustrative Example

**Figure 1:**
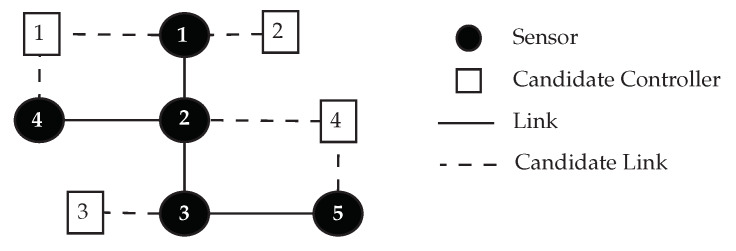
A network with four sensors and three candidate controllers.

Figure 1 illustrates a simple example to clarify our system model. In the example there are five sensors and four candidate locations to place controllers. Let us assume k=2, $l_{max}=3$, and SyncLimit for the given network is 7 Mbps. The cost of inter-controller synchronization, i.e., ${SyncCost}^{Controller}$, for using 2, 3, and 4 controllers is equal to 3 Mbps, 6 Mbps, and 9 Mbps, respectively. Apparently, in case of using only one controller, since no synchronization is needed, the ${SyncCost}^{Controller}$ is equal to 0. Therefore, according to Equation (5), the maximum number of controllers for which the ${SyncCost}^{Controller}$ does not exceed the SyncLimit is equal to 3. In this regard, only three controllers will be placed. Accordingly, all possible combinations for placing three controllers in four candidate locations are (1, 2, 3), (1, 2, 4), (2, 3, 4), and (1, 3, 4).

For each possible combination, the furthest controller to each sensor and the distance between them are calculated and listed in Table 1. Considering the network shown in Figure 1, in the first case, the distance between the sensors and their furthest controller is 3, 2, 3, 3, and 2 hops, for Sensor 1, Sensor 2, Sensor 3, Sensor 4, and Sensor 5, respectively. In the second case, it is 2, 2, 3, 3, and 1, respectively. In the third case, it is 3, 2, 3, 3, and 2, respectively. Finally, in the fourth case, it is 3, 2, 3, 3, and 2, respectively. Accordingly, the average distance between sensors and their furthest controller in each case is 2.6, 2.2, 2.6, and 2.6, respectively. Thus, in this example, the candidate locations of the second case, i.e., (1, 2, 4), are selected to place the controllers.

It is worth noting that only the controllers that cover a sensor contribute in the calculation of the furthest controller to the sensor. In other words, if there is a controller farther than $l_{max}$ from the sensor, it is not considered as the farthest controller of the sensor. For example, in the first case, i.e., (1, 2, 3), although the furthest controllers to Sensor 5 are Controller 1 and Controller 2, as their distance to Sensor 5 is 4 > $l_{max}$, none of them is considered as the farthest controller to Sensor 5.

## 4. Solution Framework

Since the node placement problem is NP-hard [40,41], the time complexity of computing the exact optimal solution is exponential, and finding the global optimum is practically intractable. To provide an acceptable compromise between the quality of solutions and the runtime, using heuristic algorithms is a promising approach. In this paper, we use the Cuckoo optimization algorithm, an evolutionary meta-heuristic algorithm that is inspired by nature [42,43].

### 4.1. Cuckoo Optimization Algorithm

Many meta-heuristic algorithms are inspired by nature and have been successfully applied in a wide range of optimization problems. One of these algorithms is Cuckoo Search (CS), which is developed by Xin-She Yang and Suash Deb in 2009 [42]. This algorithm seeks to find global optimization by imitating brood parasitism of some cuckoo species. Brood Parasitism of cuckoos means these birds never build their own nests and instead lay their eggs in the nest of other host birds, which just laid its eggs. If the host bird discovers that the egg is not its own, it will throw the egg away [44]. Otherwise, the cuckoo eggs hatch a little earlier than the host ones, and the cuckoo chicks may evict the host eggs out of the nest and this causes an increase in the cuckoo’s population. Thus, for cuckoos, finding a suitable habitat is key to survival [43,44].

The Cuckoo search algorithm is an evolutionary and memory-based optimization algorithm. The pseudo-code of this algorithm is presented in Algorithm 1. This algorithm starts from an initial set of mature cuckoos which lay some eggs. Each mature cuckoo represents a solution, and each egg represents a new solution. Note that each mature cuckoo can lay from 5 to 20 eggs within a maximum distance from their habitat, which is called “Egg Laying Radius” (ELR). *ELR* for each cuckoo is proportional to the total number of eggs, the number of the cuckoo’s eggs, the upper bound and the lower bound for the variable. The formula to calculate *ELR* is [45]:

(10) $$\begin{array}{c} ELR=\alpha \times \left(\frac{Number\;of\;Current\;Cuckoo^{\prime }s\;Eggs}{Total\;Number\;of\;Eggs}\right)\times \left(var_{hi}-var_{low}\right) \end{array}$$

**Algorithm 1:** Cuckoo optimization algorithm
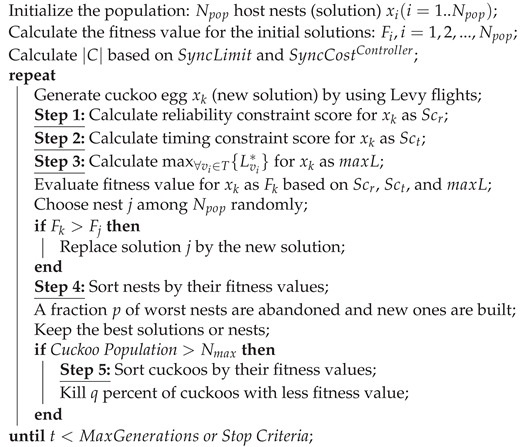


Furthermore, for each egg, a profit value is calculated that represents the probability of having the chance to grow. From all eggs, about *p* percent of them (usually 10 percent) with less profit value will be detected and killed by the host. Other eggs have the chance to grow and become a mature cuckoo, which causes an increase in the total population of cuckoos. However, since there is always an equilibrium in the bird’s population, the total population should not exceed from a maximum number called $N_{max}$. Therefore, at the end of each iteration, if the total population of mature cuckoos exceeds $N_{max}$, about *q* percent of mature cuckoos with less profit, are killed [45].

The cuckoo search algorithm has been successfully applied in several optimization problems such as job scheduling [46], production planning problem [47], and precedence constrained sequencing [48].

It is worth mentioning that, since Cuckoo search uses no gradient information during the search process, it has the ability to solve nonconvex, nonlinear, nondifferentiable, and multimodal problems [49]. It has also been successfully applied in several nonlinear optimization problems, namely in [50,51].

### 4.2. Applying Cuckoo to Our Placement Problem

As we use the cuckoo algorithm to solve the placement of controllers in WSNs, the proposed method is called Cuckoo-PC, wherein:

- Each cuckoo or each egg is a representation of *X*.
- The profit value of each cuckoo or egg is calculated according to Equation (9).
- ELR is the count of all *X* items which can be toggled from each mature cuckoo to its eggs.

Some further details regarding the framework configuration are described below:

- *Problem space:* It is the set of all possible selections from the set of candidate controllers. Therefore, each point in the problem space can be represented using an array of binary values with size $\left|A_{C}\right|$.
- *Solution representation:* Each mature cuckoo and each egg represents a point in the problem space as a potential solution.
- *Neighborhood structure:* Each mature cuckoo can lay from 5 to 20 eggs. These eggs are a subset of all reachable points by each mature cuckoo.
- *Generating a neighbor:* To generate neighbors of a point in the problem space, we use ELR, which is calculated according to Equation (10). In other words, the distance between each point in the problem space with its neighbors should be less than the ELR. Furthermore, it is assumed that the distance between two points in problem space is calculated as the number of all *X* toggled items.

## 5. Performance Evaluation

This section starts with discussing the setups adopted for the performance evaluation. Then, it presents the details of the sensitivity analysis, which is then followed by the study of the time complexity of the Cuckoo-PC algorithm. Finally, the evaluation results are depicted and discussed.

### 5.1. Experimental Setups

To conduct the experiments, we considered four benchmarks, each of which is a WSN with different sizes. The first benchmark includes 100 sensors and 16 candidate locations to place controllers and is referred to as WSN1. The second benchmark consists of 150 sensors and 22 candidate locations to place controllers and is called WSN2. The third benchmark, named as WSN3, has 170 sensors and 26 candidate locations to place controllers. Finally, the last benchmark includes 200 sensors and 30 candidate locations to place controllers and is referred to as WSN4. System parameters used in our experiments are derived from related papers, including [11,52], which are listed in Table 2. The value of SyncLimit and ${SyncCost}^{Controller}$ that are required to calculate the number of selected controllers for each benchmark in Equation (5) is set according to [32].

To evaluate Cuckoo-PC, we have implemented an optimization framework in Java that includes Cuckoo-PC, quantum annealing, and simulated annealing algorithms. The proposed optimization framework is publicly available in [53].

To run the experiments, a laptop with Mac OS, Core i5 2.9 GHz, and 8 GB memory is used. The algorithms were run 30 times to reach a 95% confidence interval.

For performance evaluation, we also define three metrics based on the optimization problem formulated in Equations (8) and (9), namely ψ, ϕ, and γ. These metrics are listed in Table 3.

### 5.2. Sensitivity Analysis

Since the algorithm parameters have a substantial impact on the performance of the Cuckoo-PC algorithm, a sensitivity analysis is performed to measure the effectiveness of every parameter. In the sensitivity analysis, a reasonable range for every parameter of the algorithm is investigated to find the best values.

**Figure 2:**
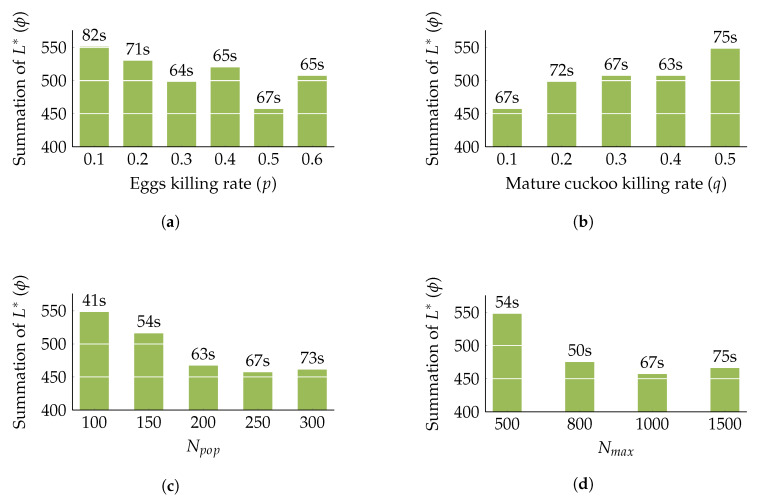
Sensitivity analysis of Cuckoo-PC parameters. All experiments are conducted on WSN3. (**a**) Sensitivity analysis of p, (**b**) Sensitivity analysis of q, (**c**) Sensitivity analysis of $N_{pop}$, (**d**) Sensitivity analysis of $N_{max}$.

In this section, to determine the proper value of the algorithm parameters, we conduct a sensitivity analysis considering all the parameters of Cuckoo-PC. The reasonable range of parameters is derived from related papers, including [46,47,48]. Then, multiple values within the specified ranges are checked to find the proper value of each parameter. The effects of varying algorithm parameters are represented in Figure 2a–d. Finally, all selected values for algorithm parameters, along with the range of parameters, are listed in Table 4.

It is worth mentioning that all the experiments reported in this section are conducted on WSN3. Additionally, in each set of experiments, excluding the parameter which is under investigation, the other parameters are set to the values listed in Table 4. For example, when the eggs killing rate (*p*) is being investigated (Figure 2a), the mature cuckoo killing rate (*q*) is set to 0.1, the number of eggs per cuckoo is randomly selected in a range from 5 to 20, $N_{pop}$ is set to 250, and $N_{max}$ is set to 1000.

**Eggs Killing Rate (*p*).** As is shown in Figure 2a, according to our experiments, when the eggs killing rate is set to 0.5, the best results can be achieved in terms of the summation of the average of $L^{*}$. Increasing the eggs killing rate higher than 0.5 does not improve the quality of the solutions (in terms of the summation of the average of $L^{*}$); it, however, prolongs the execution time of Cuckoo-PC unnecessarily.

**Mature Cuckoo Killing Rate (*q*).** The impact of *q* on the quality of the solution in terms of summation of $L^{*}$ is depicted in Figure 2b. As it is shown in this figure, when *q* increases, the quality of the solution is degraded significantly. Hence, the most efficient result is achieved when *q* is set to 0.1.

**Total Cuckoo Population ($N_{pop}$).** The number of total cuckoo population implies the number of regions in the problem space which are explored. Hence, by increasing $N_{pop}$, the chance of finding the optimal solution rises. According to our experiments, as is shown in Figure 2c, this parameter has a more considerable impact on the efficiency of Cuckoo-PC in comparison to other parameters. Moreover, it also has a prominent effect on the convergence of the algorithm.

When $N_{pop}$ goes higher, the quality of the solutions is improved at the expense of spending more execution time. The best solution is achieved when $N_{pop}$ is set to 250. However, when $N_{pop}$ is set to lower values (i.e., 100 and 150), although the average execution time of Cuckoo-PC is decreased, the quality of the solution is getting worse, revealing a premature convergence. Moreover, when $N_{pop}$ is set to a higher value such as 300, there is no considerable improvement in the quality of the solution, whereas the execution time of Cuckoo-PC dramatically rises.

**Maximum Cuckoo Population ($N_{max}$).** As is shown in Figure 2d, when the value of $N_{max}$ is set to 1000, the best solution is achieved. However, when $N_{max}$ is set to a higher value such as 1500, there is no considerable improvement in the quality of the achieved solution, whereas the execution time of Cuckoo-PC severely increases.

### 5.3. Time Complexity

Cuckoo-PC, as presented in Algorithm 1, is composed of five main steps. A brief explanation of each step and its relevant time complexity is as follows:

- Step 1: The first step is to calculate the reliability constraint score, according to Equation (3). The time complexity of this step can be analyzed as: $O\left(\left|T||A_{C}\right|\right)$.
- Step 2: The second step is to calculate the timing constraint score, according to Equation (4). This step has time complexity: $O\left(\left|T||A_{C}\right|\right)$.
- Step 3: The third step is to calculate the maximum distance between sensors and controllers, according to Equation (8). The time complexity of this step is: $O\left(\left|T||A_{C}\right|\right)$.
- Step 4: The fourth step is to sort all eggs by their fitness values. Since according to Table 4, each mature cuckoo has at most 20 eggs, the time complexity of this step can be written as $O\left(20\times N_{pop}\log_{}N_{pop}\right)$
- Step 5: The fifth step is to sort mature cuckoos by their fitness values, which its time complexity is: $O\left(N_{pop}\log_{}N_{pop}\right)$

Hence, the time complexity can be written as $O\left(N_{pop}\log N_{pop}+\left|T||A_{C}\right|\right)$. Moreover, since increasing the network size does not result in a considerable change in $N_{pop}$, it can be concluded that $\left|T||A_{C}\right|$ has dominance over $N_{pop}\log N_{pop}$. Thus, the overall time complexity of Cuckoo-PC is: $O\left(\left|T||A_{C}\right|\right)$.

### 5.4. Results

To investigate and evaluate the performance of Cuckoo-PC against other methods introduced in the literature, we use the ILP method proposed by Mousavi et al. in 2018 [30] as the baseline. The reason to opt for this method is that it is the state-of-the-art method for placing controllers in WSNs.

**Figure 3:**
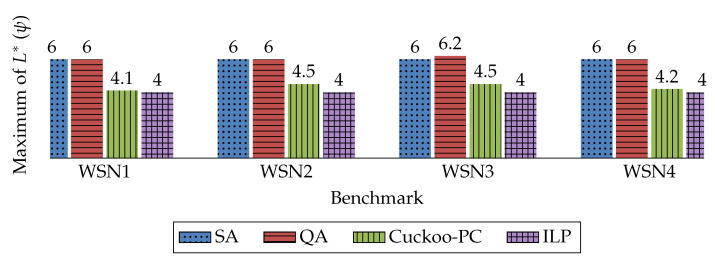
The maximum amount of $L^{*}$ between all nodes (ψ). (The average value over 30 runs of the algorithms).

**Figure 4:**
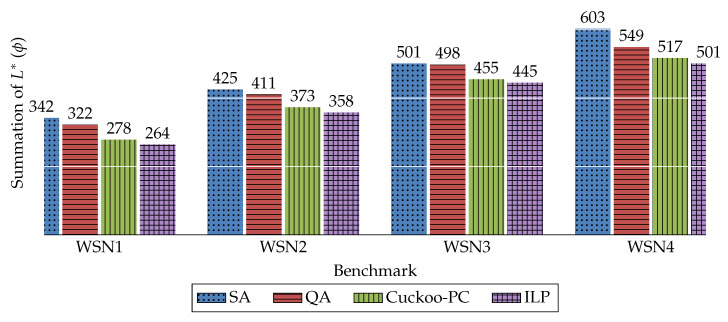
The summation of $L^{*}$ for all nodes (ϕ). (The average value over 30 runs of the algorithms).

**Figure 5:**
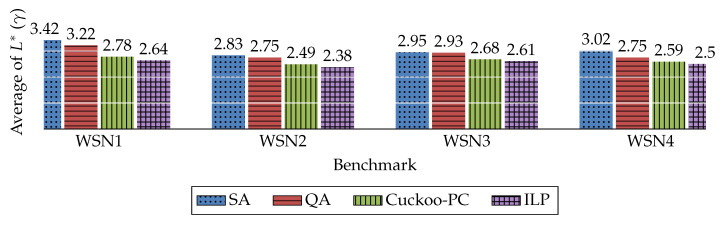
The average amount of $L^{*}$ for each node (γ). (The average value over 30 runs of the algorithms).

**Figure 6:**
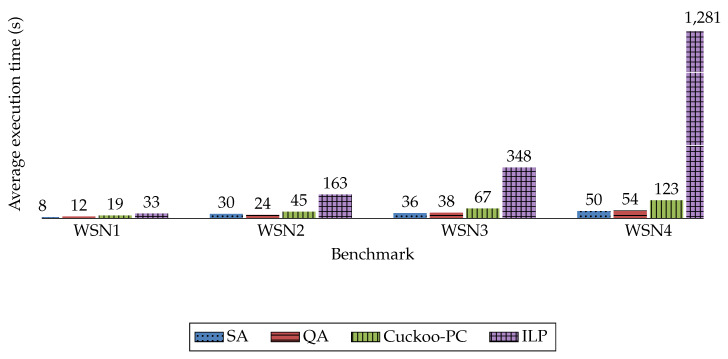
The average execution time.

The results achieved by the four methods in terms of these parameters are illustrated in Figure 3, Figure 4 and Figure 5. The comparison of the execution time of these four methods is also available in Figure 6.

Furthermore, to clearly demonstrate the performance enhancement of Cuckoo-PC against other baselines (i.e., SA and QA), Table 5 lists the percentage of improvement in ψ, ϕ, and γ, achieved by our method against SA and QA for the considered WSNs. For example, for WSN1, Cuckoo-PC outperforms SA and QA in ψ by 33%.

As shown in Figure 3 and Figure 6, and Table 5, in terms of the maximum amount of $L^{*}$ for all nodes, ILP achieves the best results for all benchmarks, whilst its execution time is much longer compared with other methods. On the other hand, Cuckoo-PC generates approximately the same results in a more reasonable time. Therefore, comparing Cuckoo-PC against ILP reveals that in large network sizes, for example, a WSN with 1000 sensors, ILP is infeasible due to its extremely long processing time, whilst Cuckoo-PC finds a near-optimal solution in a reasonable time. Furthermore, compared with QA and SA, Cuckoo-PC achieves the best results for all benchmarks, whilst QA and SA obtain almost identical results, which, on average, are about 33% worse than Cuckoo-PC.

Due to the definition of ϕ and γ in Table 3, these two parameters reflect the same aspects of the results. Thus, as shown in Figure 4 and Figure 5, and Table 5, it is concluded that in terms of the summation and the average of $L^{*}$, Cuckoo-PC outperforms the other two algorithms in all the WSNs. The improvement percentage in terms of the summation and average of $L^{*}$ of Cuckoo-PC in WSN4, against QA is about 6% and against SA is about 14%.

Moreover, considering Figure 6, although Cuckoo-PC is much faster than ILP, it is a bit slower than the other SA and QA. The reason for the larger execution time of Cuckoo-PC compared to SA and QA is inherent in the nature of population-based meta-heuristic algorithms (e.g., Cuckoo, Genetic Algorithm, Ant Colony Optimization) where multiple regions of the problem space are explored simultaneously whereas in individual-based meta-heuristic algorithms (such as SA, QA, and Tabu search) only one area of the problem space is explored at each iteration of the algorithm.

### 5.5. Interaction between SyncLimit and L^*^

In this section, we aim to profoundly investigate the effects of varying SyncLimit on $L^{*}$. To do so, we have conducted five experiments, all of them are performed on WSN3. In each experiment, all of the parameters, excluding SyncLimit, are set to values listed in Table 2 and Table 4. According to Table 2, the legitimate value of SyncLimit for WSN3 is equal to 4.675, which leads to selecting eight controllers out of 26 candidates. However, to measure the effects of varying SyncLimit on $L^{*}$, we set the amount of SyncLimit to different ratios of its legitimate value. In each experiment, the value of SyncLimit is set to $\frac{1}{3}$, $\frac{1}{2}$, 1, 2, and 3 times the amount of its legitimate value, respectively. Hence, the number of selected controllers in each experiment is equal to 4, 5, 8, 16, and 24, respectively. Detailed information for each experiment is presented in Table 6.

**Figure 7:**
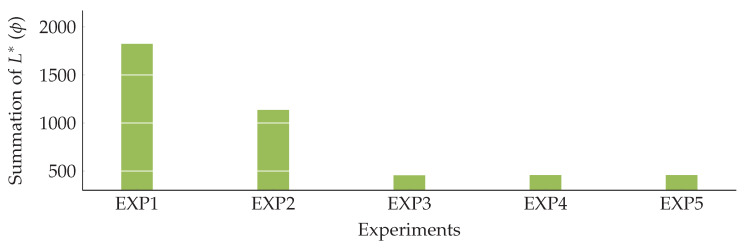
The effects of varying SyncLimit on the network performance.

The results are depicted in Figure 7. For EXP1 and EXP2, setting the amount of SyncLimit to less than the legitimate value, negatively affects the performance of the network in terms of summation of $L^{*}$. Hence, by increasing SyncLimit, there is a significant improvement in the summation of $L^{*}$. However, when the SyncLimit goes higher than the legitimate value, in EXP4 and EXP5, there is no considerable improvement in the summation of $L^{*}$.

## 6. Conclusions

Even though deploying multiple controllers can considerably improve network performance, it increases the inter-controller synchronization overhead. Therefore, it is an important research challenge to locate SDN controllers in a WSN in order to achieve maximum performance considering the constraints coming from the inter-controller synchronization overhead. We have formulated the problem as an ILP problem by considering the network performance and reliability. We also proposed the Cuckoo-PC algorithm to find a near-optimal solution in a reasonable time. To evaluate the proposed method, we compared our results in terms of the average distance between sensors and controller with other state-of-the-art methods. We considered four different benchmarks. The average improvement percentage of Cuckoo-PC against QA in terms of the average distance between sensors and controllers, is 13%, 9%, 8%, and 6% for benchmark 1, 2, 3, and 4, respectively. In terms of the average distance between sensors and controllers, Cuckoo-PC deviates from the optimal solution, generated by the ILP solver, by 5%, 6%, 2%, and 3% for each benchmark, respectively. However, the ILP method is not feasible in large-scale networks due to its extremely long execution time. The average execution time of Cuckoo-PC in the benchmarks is about 0.57, 0.27, 0.19, and 0.09 of that of the ILP method, respectively. Accordingly, the major preference to use Cuckoo-PC rather than the ILP method is that although it achieves approximately similar results as ILP, Cuckoo-PC is noticeably more scalable, which makes it a perfect solution for large scale networks. Obviously, due to the scalability issue of the ILP method, for a large-scale use case where the network may include a few thousand sensors, the ILP solver simply fails to find a solution even within a few days. That is the reason that we do not consider the ILP method as a reliable real-world solution and Cuckoo-PC is suggested.

For future work, we plan to investigate a multi-objective controller placement problem where the objectives are (i) maximizing network performance, (ii) minimizing the inter-controller synchronization overhead, and (iii) minimizing the deployment cost of the network.

## Figures and Tables

**Table 1:** Example analysis.

Combinations		Sensor 1	Sensor 2	Sensor 3	Sensor 4	Sensor 5
(1, 2, 3)	Furthest Controller	Controller 3	Controller 1	Controller 1	Controller 2	Controller 3
	Distance	3 hops	2 hops	3 hops	3 hops	2 hops
(1, 2, 4)	Furthest Controller	Controller 4	Controller 1	Controller 1	Controller 2	Controller 4
	Distance	2 hops	2 hops	3 hops	3 hops	1 hops
(2, 3, 4)	Furthest Controller	Controller 3	Controller 2	Controller 2	Controller 2	Controller 3
	Distance	3 hops	2 hops	3 hops	3 hops	2 hops
(1, 3, 4)	Furthest Controller	Controller 3	Controller 1	Controller 1	Controller 3	Controller 3
	Distance	3 hops	2 hops	3 hops	3 hops	2 hops

**Table 2:** System parameters.

Benchmarks	Sensors	*SyncLimit*	# of Candidates	# of Controllers	*k-Covered*	*Capacity*
WSN1	100	2.75 Mbps	16	5	3	30 B/s
WSN2	150	4.125 Mbps	22	7	3	30 B/s
WSN3	170	4.675 Mbps	26	8	3	30 B/s
WSN4	200	5.50 Mbps	30	10	3	30 B/s

**Table 3:** Evaluation parameters.

Parameter	Description	
ψ	Maximum amount of $L^{*}$ for all nodes	$\max_{\forall v_{i}\in T}\left\{L_{v_{i}}^{*}\right\}$
ϕ	Summation of $L^{*}$ for all nodes	$\sum_{\forall v_{i}\in T}\left\{L_{v_{i}}^{*}\right\}$
γ	Average of $L^{*}$ between all nodes	$\frac{\sum_{\forall v_{i}\in T}\left\{L_{v_{i}}^{*}\right\}}{\left|T\right|}$

**Table 4:** Algorithm parameters.

Parameter	Range	Best Value
Eggs killing rate (*p*)	0.1–0.6	0.5
Mature cuckoo killing rate (*q*)	0.1–0.5	0.1
Number of eggs per cuckoo	-	5–20
$N_{pop}$	100–300	250
$N_{max}$	500–1500	1000

**Table 5:** Improvement percentage of Cuckoo-PC against other baselines.

Benchmark	WSN1		WSN2		WSN3		WSN4	
Algorithm	SA	QA	SA	QA	SA	QA	SA	QA
ψ	33%	33%	33%	33%	33%	33%	33%	33%
ϕ	18%	13%	12%	9%	9%	8%	14%	6%
γ	18%	13%	12%	9%	9%	8%	14%	6%

**Table 6:** Amount of SyncLimit in each experiment.

	EXP1	EXP2	EXP3	EXP4	EXP5
Ratio	$\frac{1}{3}$	$\frac{1}{2}$	1	2	3
SyncLimit	1.558	2.3375	4.675	9.35	14.025
# of selected controllers	4	5	8	16	24
